# Lock or Down: Effectiveness of Isolation Measures Against COVID-19

**DOI:** 10.6061/clinics/2021/e3218

**Published:** 2021-07-26

**Authors:** Dennis Minoru Fujita, Luiz Henrique da Silva Nali, Felipe Scassi Salvador, Expedito José de Albuquerque Luna

**Affiliations:** I Laboratorio de Investigacao Medica (LIM49), Hospital das Clinicas HCFMUSP, Faculdade de Medicina, Universidade de Sao Paulo, Sao Paulo, SP, BR.; II Instituto de Medicina Tropical, Universidade de Sao Paulo, Sao Paulo, SP, BR.; III Grupo de Pesquisa de Aspectos Epidemiologicos, Clinicos, Moleculares e Celulares das Molestias Infecciosas, CNPQ/UNISA, Sao Paulo, SP, BR.; IV Programa de Pos-Graduacao em Ciencias da Saude, Universidade Santo Amaro, Sao Paulo, SP, BR.; V Centro Universitario das Americas (CAM), Sao Paulo, SP, BR.

According to the World Health Organization (WHO), in June 3, 2021, there were 171,292,827 confirmed coronavirus disease (COVID-19) cases and 3,687,589 deaths. In addition, by June 2, 2021, a total of 1,581,509,628 vaccine doses had been administered worldwide (1).

Currently, there is no specific drug for effective and final treatment. Existing vaccines may not be effective against some variants of severe acute respiratory syndrome coronavirus 2. However, vaccine uptake is still low in most countries, and we are not sure whether the available vaccines will be effective for the new variants or new ones that might come to eventually reach a herd immunity threshold (2).

According to the current WHO guidelines, non-pharmacological preventive measures are effective in reducing the incidence of COVID-19 cases and deaths. The most important of these is the lockdown, as it restricts traffic and the consequent contact of people, preventing the formation of mass gathering, which may accelerate the transmission process of this infectious disease that occurs through droplets, aerosols, and contaminated fomites (3).

The adoption of the complete lockdown process for at least 14 days, with the maintenance of only essential services and restriction of the flow of people, allowing only safety and health professionals to transit, reduced the transmission of the disease and case-fatality rate. Although it is an extreme measure to be adopted because the virus can spread again in the region, it results in lower transmission rates that can help to contain the disease burden on the local health system.

China, Japan, Germany, Australia, New Zealand, and others are some countries that have implemented this type of public health policy, although their success was achieved by combining social distancing measures with a systematic epidemiological control of infected people, including contact tracing, timely isolation of sick individuals and persons who had been in direct contact with patients, mandatory quarantine for sick people and travelers, and other preventive measures that comprise an effective public health policy against COVID-19 (Table 1).

The lockdown measure is necessary, in view of the current situation of the absence of effective drugs for treatment, epidemiological surveillance and control of the population, and vaccines that cover the spectrum of variants that are increasingly diversified, especially in countries that have been negligent in managing the disease in their territories (4).

However, the lockdown has socio-economic consequences that must be considered by government authorities when it is implemented. It is important to have an action plan to prevent vulnerable populations from being exposed, providing financial support, food, healthcare, and other basic conditions to comply with the quarantine period required during the lockdown (5).

The various segments of the economy must participate in the process and be guided and assisted by governments through public policies of fiscal incentives, lines of financing, and labor regulation, among others, to deal with economic and productivity impacts resulting from the mandatory lockdown and absence of consumption because of the instability created by the fear of the pandemic. The economic recovery after the establishment of the lockdown depends on this joint action (6).

The period of isolation from the peak of incidence to the reduction of cases for the reopening of activities, until a new period of increased incidence (reopening of activities), creates a predictable interval of three to four months that allows the resumption of the various activities as well as the economy of these locations, enabling companies and governments to plan within this new productive context.

However, some nations and regions insist on another type of social isolation, the partial lockdown, which, unfortunately, has a greater permissiveness that produces a slight effect on the incidence, as part of the population adheres to the process, although the majority, mainly the denialists, do not comply with restrictive and protective measures such as the use of masks, prohibition of mass gatherings (parties), and others (7).

The problem with this process is the impossibility of planning the economic recovery by the authorities (federal or local) and companies because of the incidence of cases and deaths remaining in continuous flow, as in Brazil, with no quantitative reduction that allows the return of general activities in adequate safety conditions, for example, the resumption of schools for face-to-face classes.

This type of measure may please companies and politicians, as it allows the economy to be in apparent operation. However, there is no way for certain segments to continue, especially if involving mass gathering, as they can generate unprecedented growth in the incidence, as has occurred in Brazil in the current context, after the various holidays at the end of the year and reduction of preventive measures adopted by some localities.

The United States (Figure 1) adopted the same negative attitude, although since the change in posture with adherence to preventive measures in 2021, including the incentive for vaccination, there has been a significant reduction in incidences and deaths from January (constant increase flow of positive cases) to March (sharp decline in the case-fatality incidence) 2021, with a chi-square curve with 56711.1 df, z of 238.1, and *p*-value<0.0001, corroborating the hypothesis that non-pharmacological measures, including social distance, are fundamental for the protection of people and for the resumption of the economy.

In this sense, we realize the inexistence of the paradigm that national economies can fail because of the lockdown (Table 2), although it can occur if there is uncertainty of economic recovery by nations and companies, where investors tend to retreat in unpredictable economic scenarios.

Unfortunately, we need to reinforce the issue that preventive measures are important and that they do not lead to the collapse the economy if properly coordinated by the authorities.

Economic collapse can occur because of the uncontrolled growth of the disease without producing herd immunity. However, the development of more contagious and lethal variants, in addition to the expenses that could be avoided, such as the treatment of patients and deaths, purchasing equipment and supplies, and days of not going to work, might not exist if there was an effective national public health policy to combat this disease.

## AUTHOR CONTRIBUTIONS

All authors affirmed that they contributed to the development of the manuscript. Fujita DM designed the study, collected data, and wrote and edited the manuscript. Nali LHS designed the study, collected data and discussed the results. Salvador FS contributed to the investigation, methodology and manuscript review. Luna EJA performed the analysis and reviewed the manuscript.

## Figures and Tables

**Figure 1:**
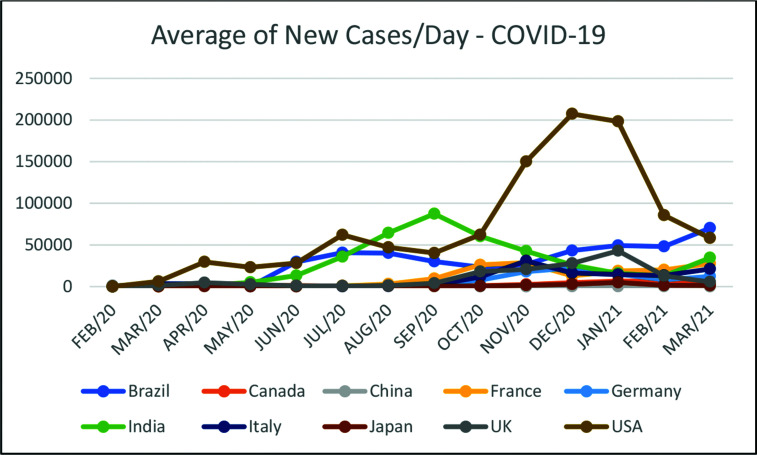
Average of New Cases/Day - COVID-19.

**Table 1:** Ranking of Countries by Total Positive Cases and Lethality.

COVID-19 - Incidence and case-fatality - 10 Main Economies - 2020/2021				
Ranking	Country	Total confirmed cases	Deaths	per 100,000 population
1	United States	32,963,138	589,555	9,958.57
2	India	28,441,986	337,989	2,061.01
3	Brazil	16,624,480	465,199	7,821.10
4	France	5,584,232	108,925	8,555.13
5	United Kingdom	4,494,703	127,794	6,620.96
6	Italy	4,223,200	126,283	6,984.90
7	Germany	3,692,468	88,940	4,407.13
8	Canada	1,383,214	25,566	3,664.90
9	Japan	752,191	13,245	594.73
10	China	112,458	4,995	7.81

Source: World Health Organization, 2021 - until June 3, 2021.

**Table 2:** Ranking of Countries by GDP—2016-2020.

GDP (USD in Trillion) - 10 main economies - 2016-2020						
Ranking	Country	2016	2017	2018	2019	2020
1	United States	18.71	19.51	20.58	21.43	20.93
2	China	11.23	12.31	13.89	14.28	14.70
3	Japan	4.92	4.86	4.95	5.08	4.91
4	Germany	3.46	3.68	3.96	3.86	3.80
5	India	2.29	2.65	2.71	2.86	2.59
6	United Kingdom	2.69	2.66	2.86	2.82	2.83
7	France	2.47	2.59	2.78	2.71	2.60
8	Italy	1.87	1.96	2.09	2.00	1.88
9	Brazil	1.79	2.06	1.88	1.84	1.83
10	Canada	1.52	1.65	1.71	1.73	1.73

Source: United Nations, World Bank, 2021.
